# Development of an affordable light emitting diode spectrophotometer paired with a Python program for calibration and linearity testing and the measurement of uranium(VI)

**DOI:** 10.1371/journal.pone.0308516

**Published:** 2024-09-17

**Authors:** Amrutaa Vibho, Courtney Rogat, Emily Karavas, Rahisa Mohammed, Peace Ogadi, Michael White, Thomas Salois, Charles Anderson, Michael W. Prairie, Seth H. Frisbie, Sarah K. Gallant

**Affiliations:** 1 Department of Chemistry and Biochemistry, Norwich University, Northfield, Vermont, United States of America; 2 Department of Computer Science, Colorado State University, Fort Collins, Colorado, United States of America; 3 Department of Electrical and Computer Engineering, Norwich University, Northfield, Vermont, United States of America; Khalifa University of Science and Technology, UNITED ARAB EMIRATES

## Abstract

Uranium (U) is a radiologically and chemically toxic element that occurs naturally in water, soil, and rock at generally low levels. However, anthropogenic uranium can also leach into groundwater sources due to mining, ore refining, and improper nuclear waste management. Over the last few decades, various methods for measuring uranium have emerged; however, most of these techniques require skilled scientists to run samples on expensive instrumentation for detection or require the pretreatment of samples in complex procedures. In this work, a Schiff base ligand (P1) is used to develop a simple spectrophotometric method for measuring the concentration of uranium (VI) with an accurate and affordable light-emitting diode (LED) spectrophotometer. A test for a higher-order polynomial relationship was used to objectively determine the calibration data’s linearity. This test was done with a Python program on a Raspberry Pi computer that captured the spectrophotometer’s calibration and sample measurement data.

## Introduction

As the demand for alternative power sources, including nuclear energy, increases, the need for facile testing methods for uranium (U) in the environment also rises. Uranium can leach from geological materials into groundwater sources due to natural processes and anthropogenic operations, including uranium mining and processing for nuclear power and improper nuclear waste management [1]. The most common cause of exposure to uranium is through ingestion of water or food which have been contaminated. Although radiological toxicity may cause adverse health effects, the primary human health concern is due to the acute and long-term chemical toxicity of uranium in the human body [2].

The primary exposure pathway to uranium has been reported as “direct ingestion of well water,” which makes up 99% of the toxicological and radiological doses to human consumers [3]. According to the “Public Health Statement for Uranium” from the Agency for Toxic Substances and Disease Registry (ATSDR), uranium enters the bloodstream via the gastrointestinal tract (mouth, stomach, intestines). Water-soluble uranium compounds can more easily enter the bloodstream than compounds that do not dissolve well, resulting in the most severe systemic effects. Absorbed uranium is processed through the kidneys and leaves the body in urine but may be deposited in the bones, liver, or kidneys for weeks [4]. Long-term exposure to uranium can result in kidney disease, kidney failure, hypertension, osteoporosis, or cancer [2, 5].

An important factor about uranium consumption from food, air, or water is that if the source of exposure is removed, renal damage will normally reverse naturally, so early testing is key [6]. Unfortunately, uranium is toxic even at relatively low levels. The World Health Organization (WHO) guideline for uranium in drinking water is 30 micrograms per liter (μg/L); however, certain populations, such as young children or people with predispositions to hypertension, osteoporosis, or kidney disease, may be at greater risk with uranium exposure [5]. Currently, there is no accurate, safe, and inexpensive test to measure uranium in drinking water. Economically disadvantaged people from across the world could utilize such a test, including in the United States, where the Navajo Nation is exposed to uranium from mining operations [7]. In addition, large areas of the Himalayan drainage basin, such as northern India, Bangladesh, and Myanmar, are exposed to unsafe concentrations of uranium in their drinking water and would benefit from a safe, accessible, and inexpensive test [5, 8–12].

Uranium is typically detected using expensive equipment operated by a specialist. The current state of the art is quadrupole-based inductively coupled plasma-mass spectrometry (ICP-MS) [13–16]. Although some methods for spectrophotometric determination have recently been reported, they often involve preconcentration or pretreatment, such as flow-injection, acidification, or microextraction, which require specialized or custom-built equipment [17–19].

To meet the growing need for a simple, inexpensive test for uranium that can be completed by a layperson, in this article, we demonstrate the use of a $63 United States dollars (USD) light-emitting diode (LED) spectrophotometer previously reported to accurately assay the presence of iron, manganese, and fluoride [20] to detect uranium.

## Materials and methods

### Reagents and instruments

All starting materials were purchased from commercial suppliers. Rhodamine 6G and 2-hydroxy-1-naphthaldehyde were purchased from Sigma Aldrich, 80% hydrazine from Tokyo Chemical Industry Co., Ltd. (TCI), a subsidiary of VWR International (VWR), and uranyl nitrate hexahydrate from Thermo Fisher Scientific Inc. Solvents were American Chemical Society (ACS) reagent grade and were purchased from VWR. The P1 ligand (Schiff base 3-(3,6-bis(ethylamino)-*9H*-xanthen-9-yl)-2-(((2-hydroxynaphthalenyl)methylene)amino)isoindolin-1-one) was prepared as described previously in the literature [21].

The spectra and absorbances of the P1/U(VI) complex at 525 nanometers (nm) were measured with a Shimadzu UV-2600i ultraviolet-visible (UV-Vis) spectrophotometer. The absorbances of the P1/U(VI) complex were also measured at 525 nm with our LED spectrophotometer using an LED purchased from Thorlabs, Inc. Our LED spectrophotometer’s design, construction, and use were previously reported in PLOS ONE [20]. The analog voltage from our LED spectrophotometer was digitized with MCP3008 8-Channel, 10-bit analog to digital converter from Microchip Technology Inc. The digital voltage was sent to a Raspberry Pi 3 purchased from Adafruit Industries. This digital voltage was processed with our Python-language computer program, which is described here for the first time. Our Python code is included in the Supporting Information (S1 File).

### Ultraviolet-visible spectra and measurements

A 200 micromolar (μM) stock solution of P1 ligand was prepared in acetonitrile. Uranyl nitrate solutions were prepared by serial dilution of a 50,000 μg/L uranyl nitrate stock solution in acetonitrile. Finally, 1.5 milliliters (mL) of P1 stock and 1.5 mL of uranyl nitrate solution were mixed thoroughly to form standard solutions. These standard solutions were used for calibration.

### Objectively determining the linearity of the calibration data

A test for a higher-order polynomial relationship was used to objectively determine the calibration data’s linearity. The Beer-Bouguer-Lambert law, more commonly called Beer’s law, indicates that a linear calibration curve is expected. Therefore, a regression of absorbance on the concentration of uranium and the concentration of uranium squared was used to test for the significance of a quadratic effect [20]. At alpha (α) = 0.05, if the quadratic effect was not statistically significant, the calibration curve was linear at the 95% confidence level [22]. This test was done with the Python program on the Raspberry Pi computer that captured the spectrophotometer’s calibration and sample measurement data. The output of the Python program was independently confirmed with Microsoft^®^ Excel^®^ and the statistical program R.

### Calculating detection limit

In this case, the detection limit is the concentration of uranium that can be measured and reported with 99% confidence that its concentration is greater than zero. More specifically, the detection limit is the 1-tailed 99% confidence cutoff value of the measured concentrations of seven different reagent blanks [22].

### Statistical analyses

Statistical analyses were performed using R version 4.3.0 (2023-04-21 ucrt) “Already Tomorrow.” These analyses were confirmed using Microsoft^®^ Excel^®^ for Microsoft 365, version 2302, build 16.0.16130.20298. The raw data and statistical analyses are included in the Supporting Information (S2 and S3 Files).

## Results and discussion

### Design of light emitting diode spectrophotometer coupled with Raspberry Pi for data processing

**Fig 1:**
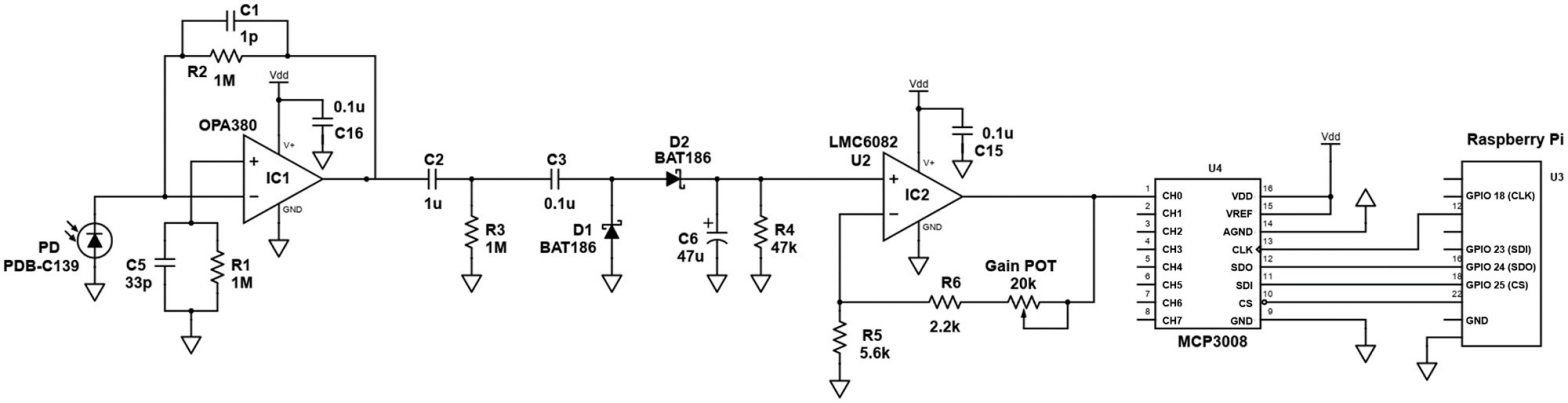
A schematic diagram of the connectivity between the detector circuit of the Light-Emitting Diode (LED) spectrophotometer, MCP3008 integrated circuit, and Raspberry Pi 3 computer (the device power and LED drive circuits are presented in [20]).

The LED spectrophotometer’s design, construction, and use were previously reported in PLOS ONE [20]. The previously reported LED spectrophotometer was an analog instrument. The readout of this instrument was a voltage displayed on a voltmeter that the user needed to convert to an absorbance value before calculating a concentration [20]. For this paper, the analog voltage from our LED spectrophotometer was digitized with MCP3008 2.7 volt, 8-Channel, 10-bit analog to digital converter. The digital voltage was sent to a Raspberry Pi 3 (Fig 1), which was then processed with our Python-language computer program. Our Python code is included in the Supporting Information (S1 File).

**Fig 2:**
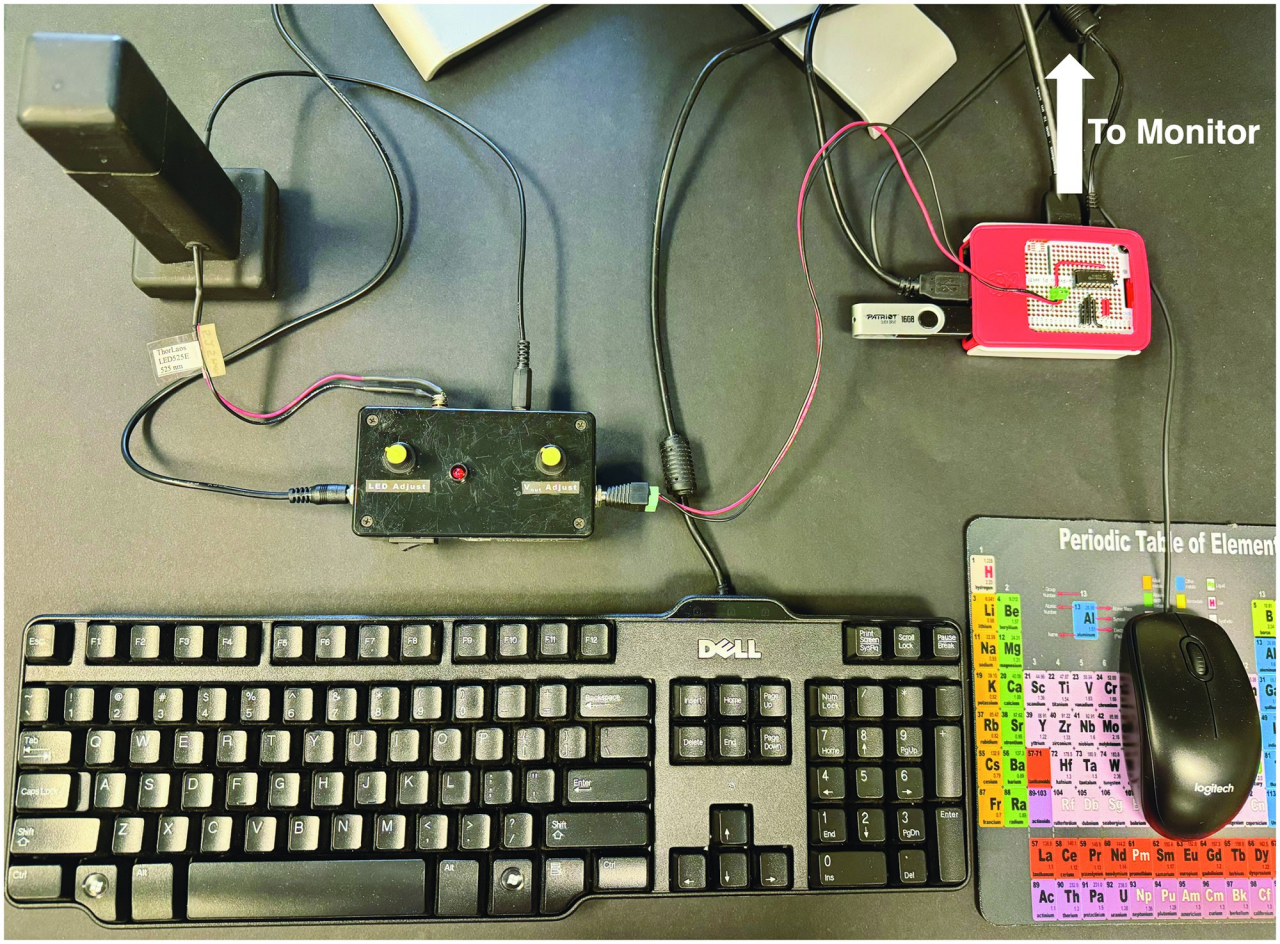
A picture of the $63 spectrophotometer coupled with a Raspberry Pi for data processing.

Combining an affordable spectrophotometer with the Raspberry Pi computer (Fig 2) allows for a user-friendly system that a layperson can use to easily calibrate the instrument and measure the analyte concentrations of samples. More specifically, the MCP3008 integrated circuit converts the analog voltages from the spectrophotometer to digitized voltages. These digitized voltages are sent to the Raspberry Pi computer. The Python code stored on the Raspberry Pi captures these digitized voltages for all the calibration standards and samples. These digitized voltages are sampled, averaged, and converted to absorbances. For each reading, 32 sampling events are collected and averaged. A digital voltage is read every 0.02 seconds, so the 32 readings take 0.64 seconds to collect. The absorbances calculated by the Python program on the Raspberry Pi are then further analyzed for linearity and the significance of the y-intercept.

The absorbances from the calibration standards are tested for a higher-order polynomial relationship to objectively determine if the calibration curve is linear or non-linear at the 95% confidence level. Then, the resultant calibration curve is tested to objectively determine if the y-intercept goes through the origin (0,0) or does not go through the origin at the 95% confidence level. The results from these two statistical tests objectively give the best-fitting calibration curve. This best-fitting calibration curve is used to convert the absorbances from the samples to analyte concentrations. Finally, these analyte concentrations are displayed to the user on a monitor and are stored in a spreadsheet on the Raspberry Pi.

Using this system, a layperson can run calibration standards and determine if the calibration curve obeys Beer’s law; that is, if it is linear and goes through the origin. If not, the user is notified and asked to check for errors and repeat the calibration procedure. The results of a valid calibration are used to calculate the concentration of analyte in unknown samples. No manual calculations or prior knowledge are required.

### Synthesis of P1 ligand, proposed mechanism of binding and resulting color change

**Fig 3:**
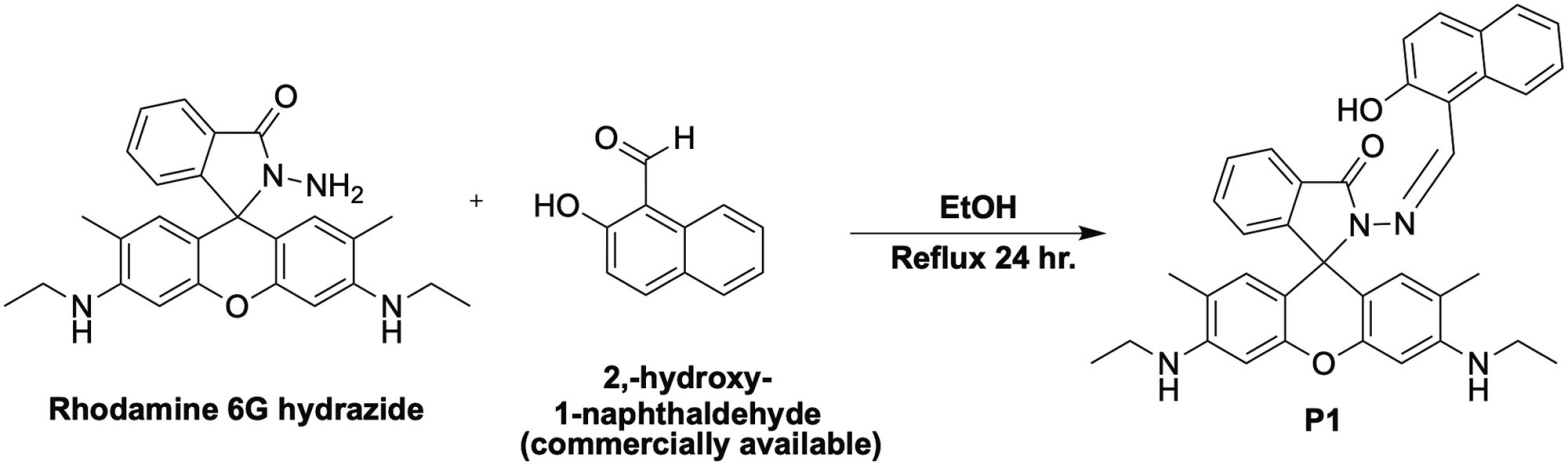
Synthesis of P1 ligand using commercially available materials.

P1 ligand (3’,6’-bis(ethylamino)-2-(((2-hydroxynaphthalen-1-yl)methylene)amino)-2’,7’-dimethylspiro[isoindoline-1,9’-xanthen]-3-one) was synthesized as described in Fig 3 [21].

**Fig 4:**
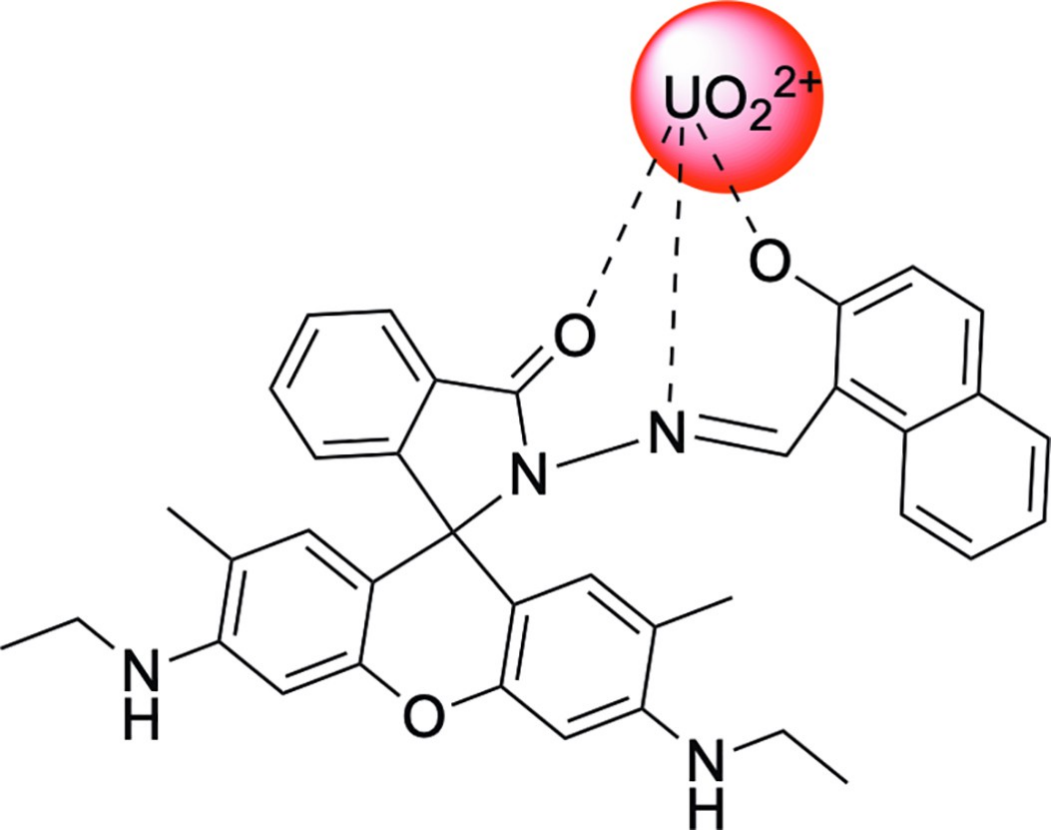
Proposed binding of uranyl (UO_2_^2+^) to P1 ligand.

The proposed mechanism of metal-ligand binding has been described previously in the literature for magnesium ion (Mg^2+^), zinc ion (Zn^2+^), gallium ion (Ga^3+^), and uranyl ion (UO_2_^2+^) [21, 23, 24]. The mechanism proposed utilizes the carbon-oxygen double bond (C = O) and carbon-nitrogen double bond (C = N) moieties, as well as coordination at the alcohol on the naphthalene ring (Fig 4), and has been investigated both experimentally and computationally [21, 23]. In this assay, the neutral pH of 7.00 required for sample preparation removes potential interfering metal ions, such as ferrous (Fe^2+^) and manganous (Mn^2+^) ions, by precipitation. This improves the method’s selectivity. In addition, prior work in this ligand system has demonstrated strong selectivity towards uranium in competition with other metal ions commonly present in water [21], further improving this method’s selectivity.

**Fig 5:**
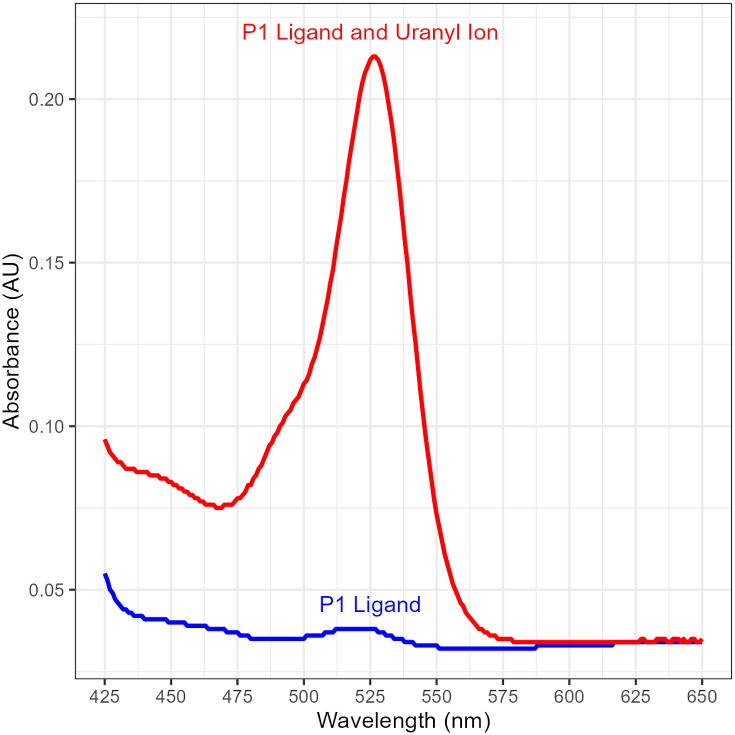
The ultraviolet-visible (UV-Vis) spectra of P1 ligand (blue) and the P1-uranyl ion complex (red).

After metal-ligand binding, a color change occurs with maximum absorbance wavelength at 525 nm. This color change arises from the breaking of the spirolactam ring in the P1 ligand [21]. Fig 5 demonstrates the change in absorbance upon binding of the uranyl ion.

### Calculating the upper limit of the linear range

Sequential tests for higher-order polynomial relationships were used to objectively calculate the upper limit of the linear range [20, 22]. A regression of absorbance on the concentration of uranium and the concentration of uranium squared was used to test the significance of a quadratic (second-order) effect [20, 22]. If this quadratic term was significant statistically at α = 0.05, the calibration equation is not a line at the 95% confidence level, as indicated by Beer’s Law [20, 22]. In contrast, if this quadratic effect is not statistically significant, the calibration curve is linear [20, 22].

To test for a quadratic effect, at least four different concentrations of standard solution are required [25]. Therefore, trials were run using the standard solutions from the four lowest concentrations and tested for a significant quadratic effect. If we obtained a statistically insignificant curve, resulting in a linear fit, we repeated this process with the absorbances from the five lowest concentrations of standard solutions. This process was repeated until we obtained a statistically significant quadratic effect [20, 22].

**Fig 6:**
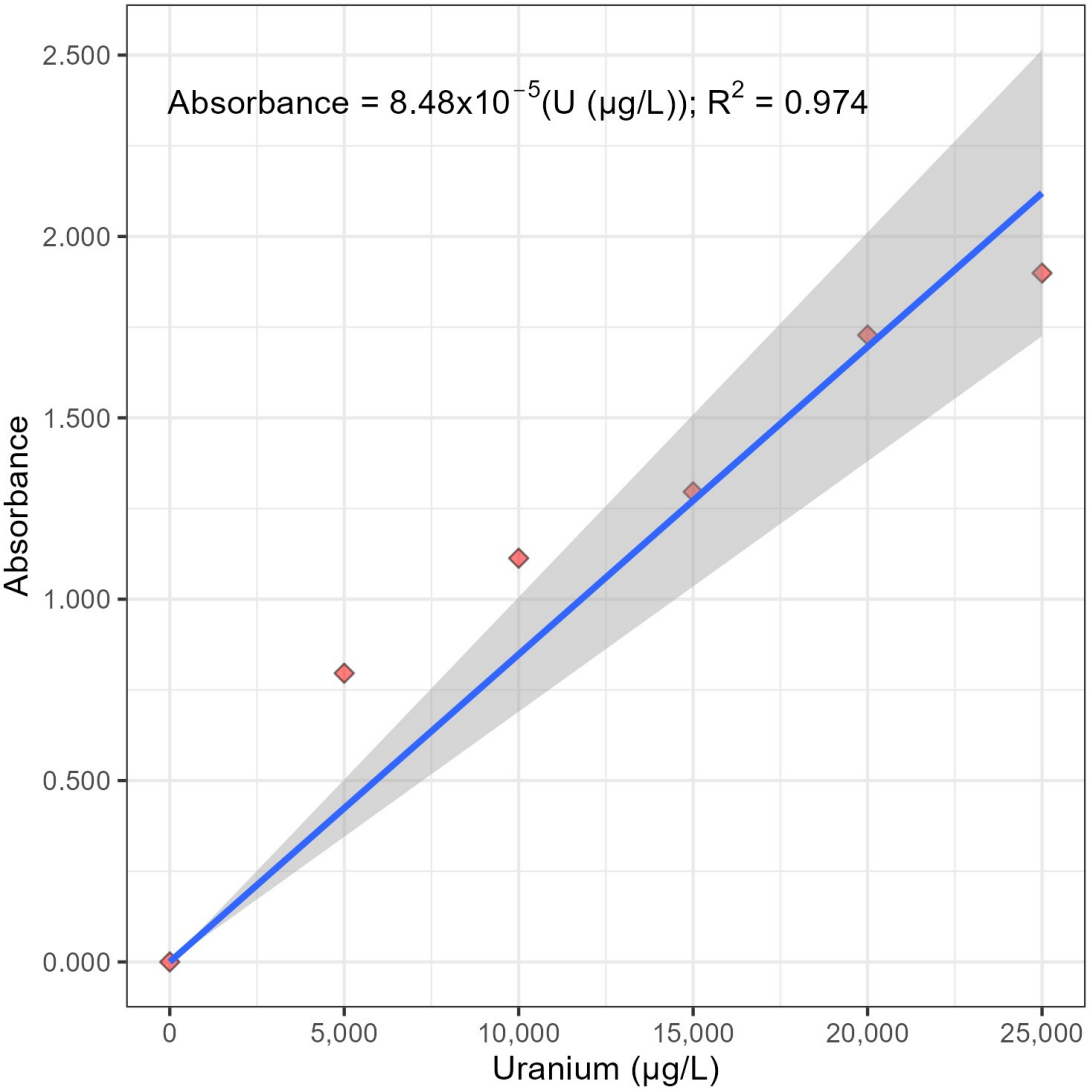
A plot of absorbance versus concentration from 5,000 to 25,000 μg/L uranyl nitrate at 525 nm.

In our study, the absorbances from 0, 5,000, 10,000, and 15,000 μg/L standards (number of samples = n = 4) give a linear calibration curve; that is, the quadratic effect was not statistically significant at the 95% confidence level (probability value = *p*-value = 0.16). Similarly, the absorbances from 0, 5,000, 10,000, 15,000, and 20,000 μg/L standards (n = 5) give a linear calibration curve. The quadratic effect was not statistically significant at the 95% confidence level (*p*-value = 0.29). Finally, the absorbances from 0, 5,000, 10,000, 15,000, 20,000, and 25,000 μg/L standards (n = 6) give a linear calibration curve (Fig 6); that is, the quadratic effect was not statistically significant at the 95% confidence level (*p*-value = 0.13). Therefore, the upper limit of the linear range for this method with this spectrophotometer is at least 25,000 μg/L. This statistical analysis can be accessed in the Supporting Information (S2 and S3 Files).

Above 25,000 μg/L uranium, a significant quadratic effect is expected, due to detector saturation [26]. For the remainder of the experiments, the concentrations were reduced to below 2,500 μg/L to ensure linearity and to determine the limit of quantification.

### The calibration curve

According to Beer’s law, at relatively low uranium concentrations, a linear calibration curve is expected, in addition to the y-intercept passing through the origin (0, 0) [20, 22, 27]. At relatively high uranium concentrations, little or no light from the source passes through and reaches the detector, resulting in a flattening of the calibration curve (Fig 6). Therefore, it is essential to know the linearity and linear range for the spectrophotometer, in addition to the upper limit, which historically was determined subjectively by visual inspection [20].

**Fig 7:**
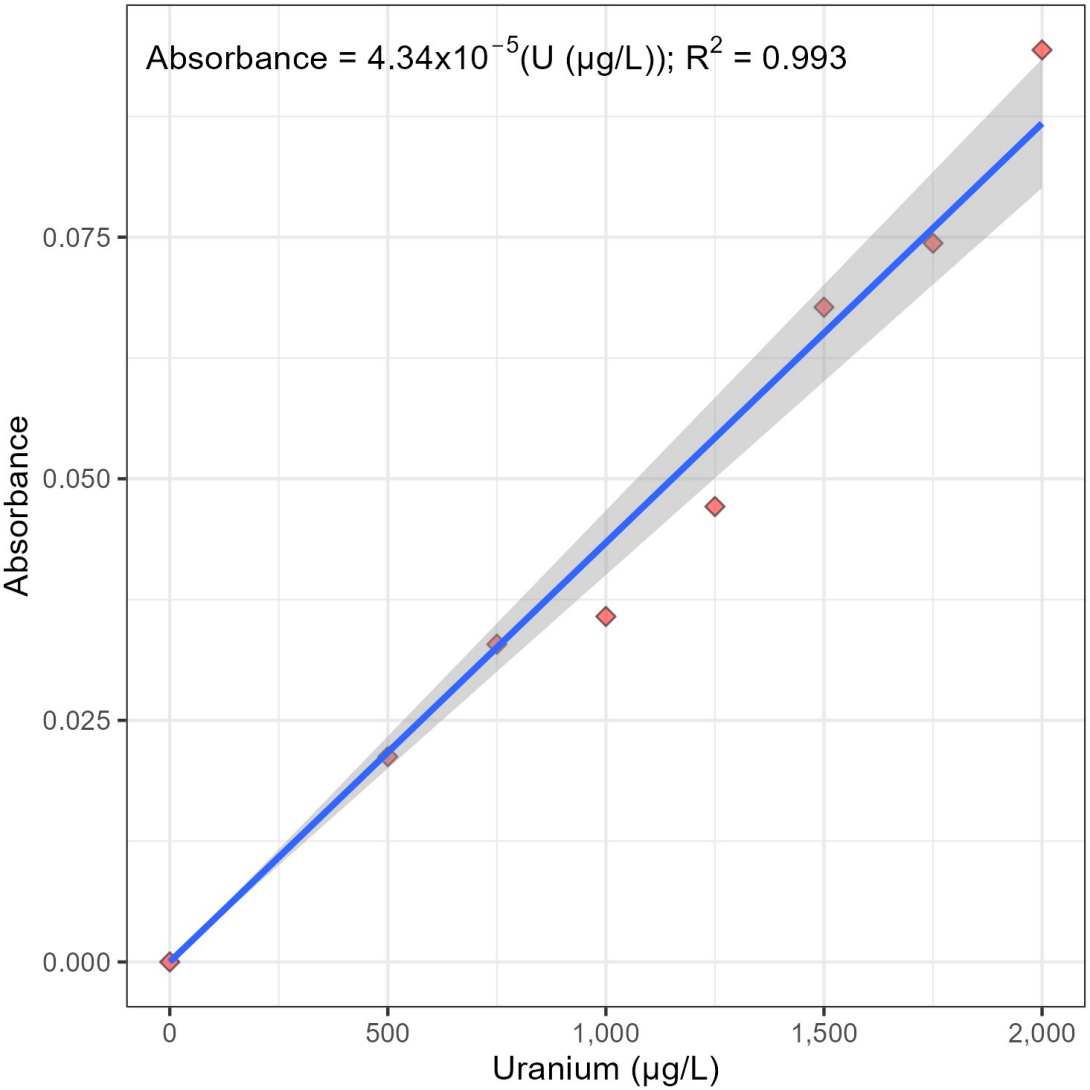
Beer’s law plot of absorbance versus concentration of uranyl nitrate in μg/L at 525 nm. Absorbances reported are an average of eight trials.

A sequential test for a higher-order polynomial relationship was used to objectively calculate a statistically significant linear calibration curve that goes through the origin, a calibration curve that obeys Beer’s law (Fig 6). More specifically, the calibration curve shown in Fig 7 does not have a statistically significant regression of absorbance on the uranium concentration and the uranium concentration squared at α = 0.05; that is, the second-order or quadratic effect is insignificant at the 95% confidence level. However, the calibration curve shown in Fig 6 does have a statistically significant regression of absorbance on the concentration of uranium at α = 0.05; that is, the first-order or linear effect is significant at the 95% confidence level. In addition, the linear regression does not have a statistically significant y-intercept at α = 0.05; that is, the y-intercept is not different than the origin at the 95% confidence level. Therefore, the final calibration curve obeys Beer’s law and was objectively calculated using linear regression through the origin (Fig 7).

The concentration of calibration standards in Fig 7 and Table 1, Experiment 1 were 0, 500, 750, 1,000, 1,250, 1,500, 1,750, and 2,000 μg/L. To investigate the robustness of the method, additional trials were performed using standard concentrations of 0, 800, 1,200, 1,600, 2,000, and 2,400 μg/L (Table 1, Experiments 2 and 3). The calibration sensitivity and R^2^ of each calibration curve are reported in Table 1. Calibration sensitivity is equal to the slope of the calibration equation. Calculations and data are reported in Supporting Information (S2 File).

**Table 1:** Calibration sensitivity of multiple calibration curves.

Experiment	Calibration Sensitivity (L/μg)	R^2^
1	4.34 x10^-5^	0.993
2	7.44x10^-5^	0.997
3	8.47x10^-5^	0.985

Experiment 1 had a lower concentration high standard (2,000 μg/L) than Experiments 2 and 3 (2,400 μg/L). Despite this, the calibration sensitivities for Experiments 2 and 3 in Table 1 are slightly larger than that for Experiment 1. In addition, the R^2^ values for Experiments 2 and 3 bracket that of Experiment 1. This suggests that the method is very robust and highly reproducible.

### Calculating the limit of detection

The limit of detection in analysis, which is often expressed as a concentration, is the least detectable measurement possible with reasonable certainty [20, 28]. The limit of detection can be derived using a variety of methods [29]. For this study, a 1-tailed 99% confidence interval was used to estimate the limit of detection from the seven separately prepared 0.00-μg/L uranium standards that were analyzed as samples (Table 2). This approach is called the method detection limit based on control charts [24]. The variability of the measured signal is centered near zero and must range from negative to positive values due to electrical noise and variability in the position of the cuvette in the optical path when the cuvette is placed into the sample holder. Based on this analysis, limit of detection for the determination of uranium with this LED spectrophotometer is 107 μg/L (Table 2). This calculation is included in the Supporting Information (S2 and S3 Files).

**Table 2:** Calculating the limit of detection based on control charts.

Label	Micrograms of Uranium per Liter (μg of U/L)
Blank 1	0
Blank 2	-25
Blank 3	100
Blank 4	0
Blank 5	-150
Blank 6	75
Blank 7	-225
Upper 99% confidence limit; this equals the detection limit based on control charts	107

### Determining the concentrations of uranium in samples

Six samples of known uranium concentration were prepared in the laboratory. These six samples were prepared using a measured mass of solid and dried uranyl nitrate (UO_2_(NO_3_)_2_) to the nearest 0.1 milligram (mg), dissolved in acetonitrile, and made to final volume using class A glassware. The final uranium concentrations of these six samples were 500, 750, 1,200, 1,250, 1,500, and 2,000 μg/L. These six actual concentrations of uranium in μg/L were compared to the associated six measured concentrations of uranium in μg/L using a paired t-test at α = 0.05. The p-value for this two-tailed paired t-test is 0.68; therefore, the actual and measured concentrations of uranium for these six samples are not significant at the 95% confidence level (Table 3). The percent (%) error was calculated as follows (Eq 1). This calculation is included in the Supporting Information (S2 File).


$$\begin{array}{c} \text{Percent}\\ \text{Error} \end{array}=\left(\frac{\;\left(\begin{array}{c} \text{Measured}\\ \text{Concentration} \end{array}-\begin{array}{c} \text{Actual}\\ \text{Concentration} \end{array}\right)}{\begin{array}{c} \text{Actual}\\ \text{Concentration} \end{array}}\right)\times 100\text{\%}$$ (1)

**Table 3:** Determining the concentrations of uranium in samples.

Actual Concentration (μg/L)	Measured Concentration (μg/L)	% Error
500	505	1
750	961	28
1,200	1,212	1
1,500	1,493	0
1,750	1,697	−3
2,000 A	1,939	−3
2,000 B	2,032	2

Based on the above data, concentrations of uranium in unknown samples can be quantified in real-time with a Python program and this portable and inexpensive LED spectrophotometer.

## Conclusion

An accurate, precise, and affordable method for measuring the concentration of uranium with an LED spectrophotometer coupled with a Raspberry Pi computer and a very powerful open-source Python program has been described. The linear range’s upper limit is at least 25,000 μg/L of uranium. The limit of detection is 107 μg/L of uranium.

## Supporting information

S1 File Python code. (DOCX)

S2 File Raw data and independent statistical analyses. (XLSX)

S3 File The R code that was used to analyze the data in S2 File. (R)
