# Supplementary material for: Development of an affordable light emitting diode spectrophotometer paired with a Python program for calibration and linearity testing and the measurement of uranium(VI)
Source: PLoS One. 2024 Sep 17;19(9):e0308516. doi: 10.1371/journal.pone.0308516 (PMC11407612; doi:10.1371/journal.pone.0308516)
Supplement: S1 File — (DOCX) [file pone.0308516.s001.docx]

# Summary of Python Code Files for Raspberry Pi

Our python code runs on a Raspberry Pi to obtain voltage values from the spectrophotometer and for selecting the calibration model and using it on sets of measurements. The code is contained in five python script files:

- spectrophotometer.py,
- model.py,
- sensor.py,
- table.py, and
- usb.py.

All are publicly available in [this Github repository](https://github.com/chuckwanderson/spectrophotometer).

The use of this python code is illustrated in the following screenshots. Following the illustration, the purposes of the code in these five python script files are summarized.

## Illustration of Use

After installing the src/*.py and necessary packages onto the Raspberry Pi, launch the code with python spectrophotometer.py. The following window will appear


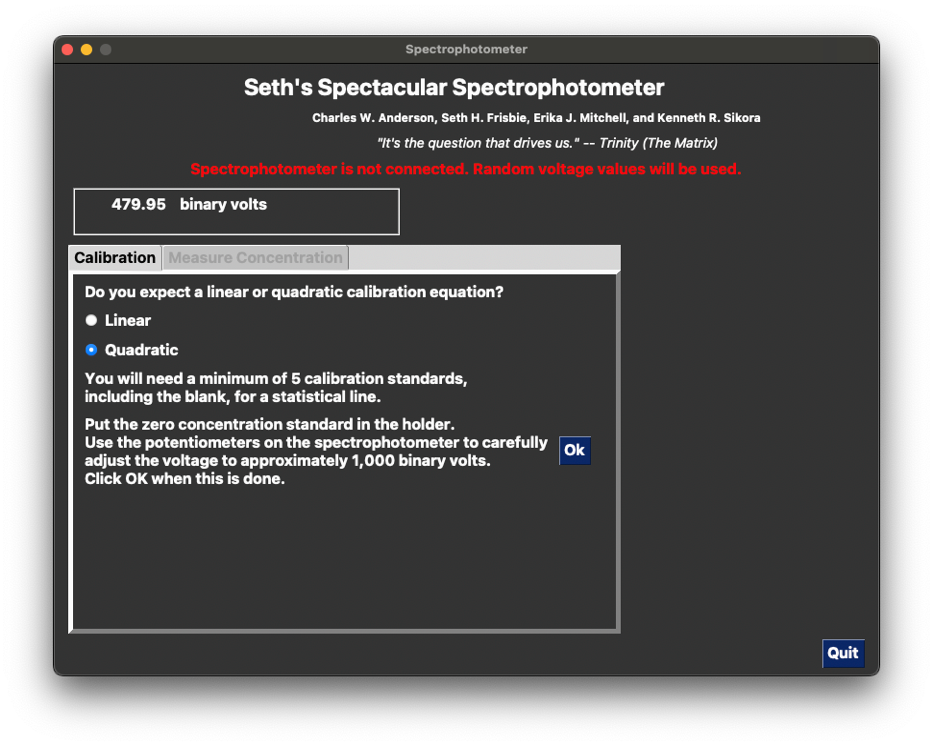


| Figure S1. The initial window. The text in red, “Spectrophotometer is not connected. Random voltage values wil be used,”, appears if the Raspberry Pi is not connected to the spectrophotometer as is the case for this illustration. |
| --- |

After clicking on OK you will go to a window in which you can record sensor readings from several samples. When done, this window will appear.


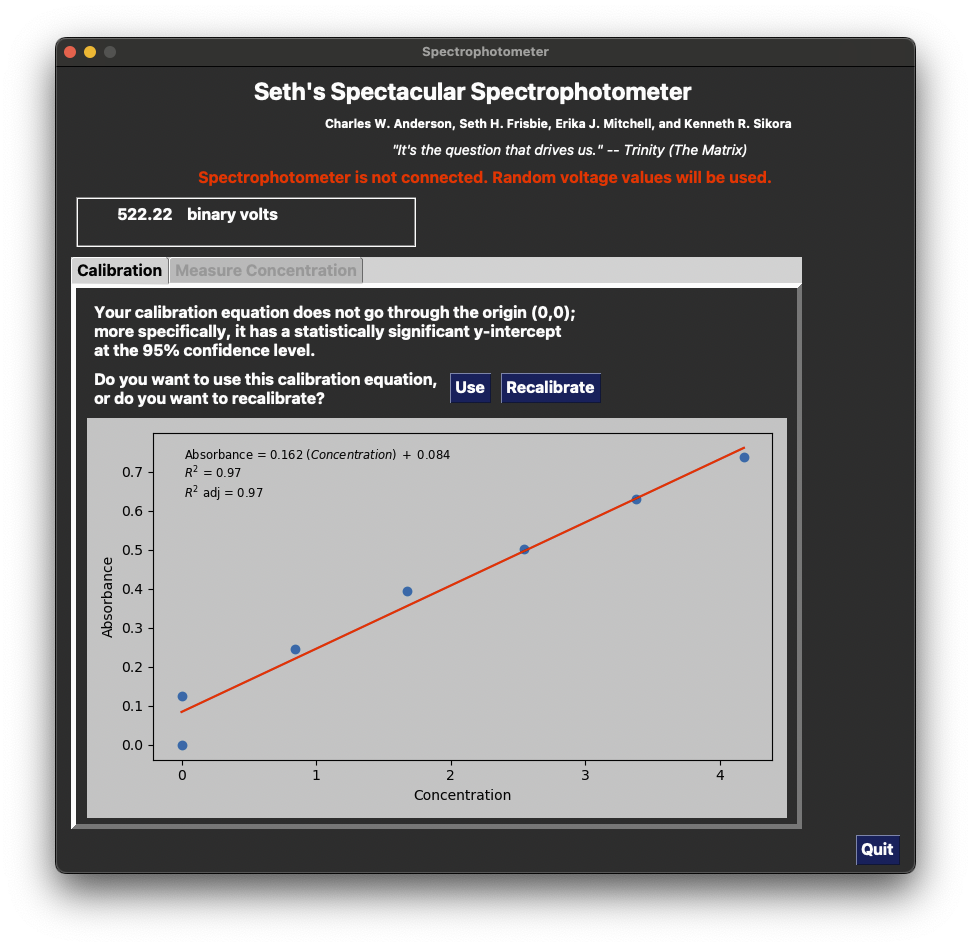


| Figure S2. A linear model with intercept has been determined to provide a statistically significant fit to the data. |
| --- |

If you choose to “Use” this calibration equation, you will advance to the following screen where you can type a sample ID, place the sample in the spectrophotometer, and click “Record”. The result will be entered in the table. When done, you can insert a USB drive into the Raspberry Pi and save this table into a csv file on the drive.


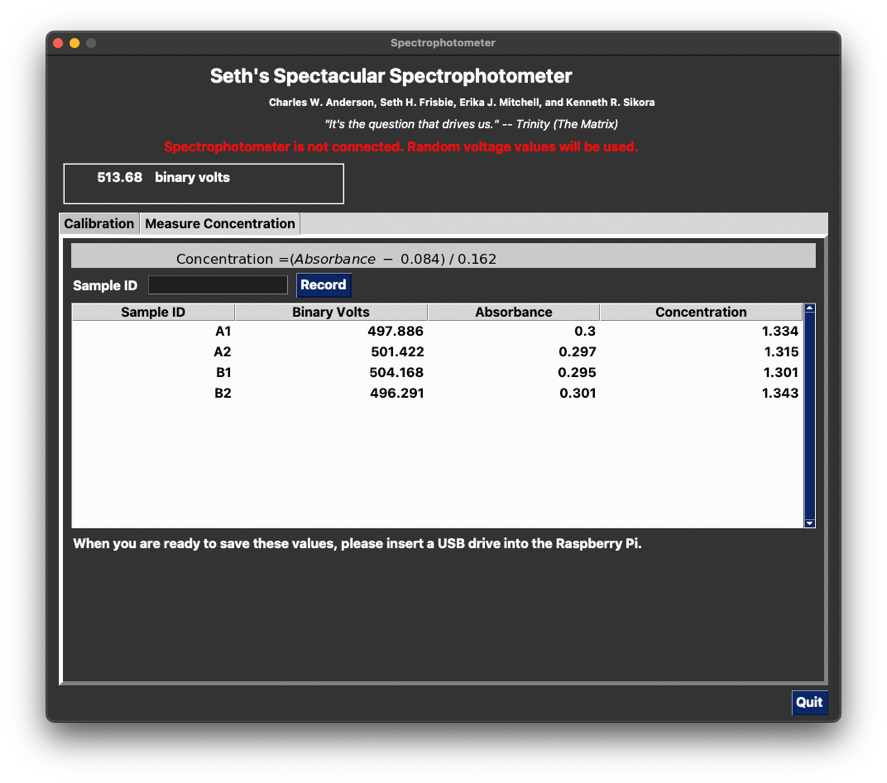


| Figure S3. The linear model is used to measure the concentration of four samples. |
| --- |

If your initial calibration samples are not fit well with a linear or quadratic formula, a cubic formula is tried. Here is an example.


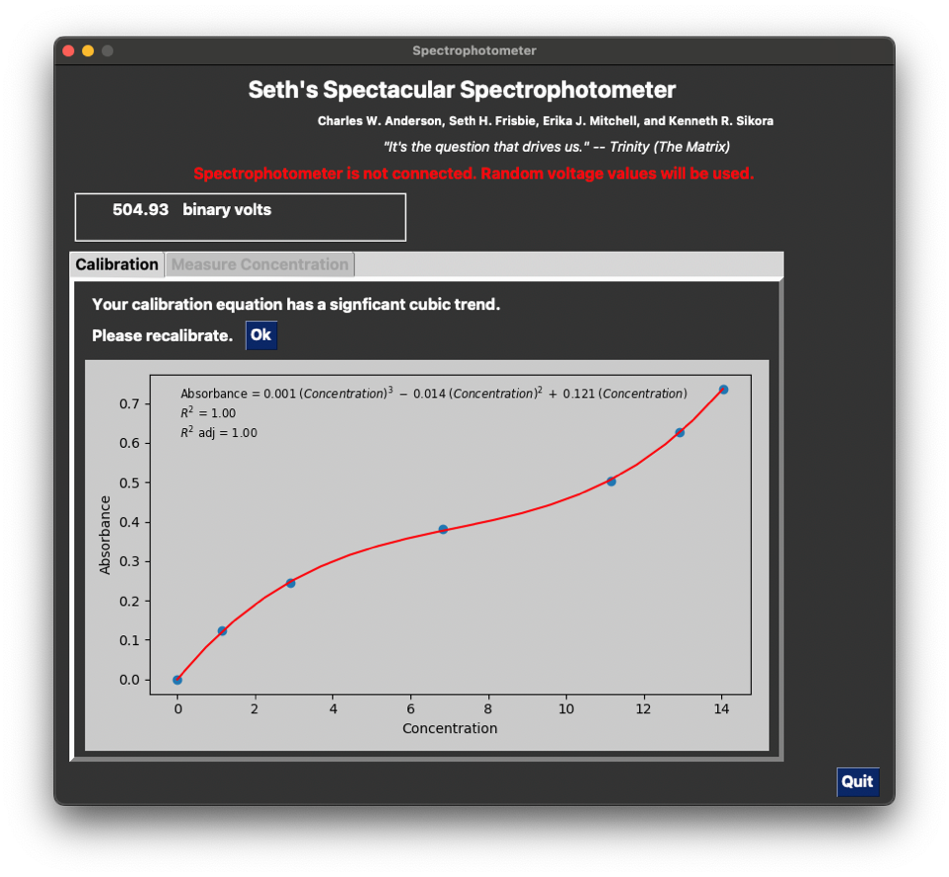


| Figure S4. A cubic model is calculated for these samples. |
| --- |

## Summaries of Python Code Files

### spectrophotometer.py

This code creates the layouts of all screens and defines functions that will be run based on the user’s choices through their interactions with the user interface text boxes and buttons.

This code also initiates the main loop. Each iteration of this loop consists of responding to all events from the user interface components by calling the appropriate functions, and reading and displaying the current voltage from the spectrophotometer circuit (see sensor.py).

This file also defines the critical function done_recording. This is the function that implements the procedure for checking the significance of the fit to the recorded data of the polynomial of the desired degree, plots the data and polynomial function values, and asks the user to confirm the resulting model.

If quadratic polynomial is wanted:
 Fit recorded data with cubic polynomial.
 If cubic trend is significant:
 Plot data with cubic polynomial values.
 Tell user to "Please recalibrate".
 If cubic trend is not significant:
 Fit recorded data with quadratic polynomial.
 If quadratic trend is significant:
 Plot data with quadratic polynomial values.
 If y-intercept value is significantly non-zero:
 Report result to user and ask if they wish to
 use this model or recalibrate.
 If y-intercept value is significantly zero:
 Report result to user and ask if they wish to
 use this model or recalibrate.
 If quadratic trend is not significant:
 Fit recorded data with a linear polynomial.
 If linear trend is significant:
 Plot data with linear polynomial values.
 If y-intercept value is significantly non-zero:
 Report result to user and ask if they wish to
 use this model or recalibrate.
 If y-intercept value is significantly zero:
 Report result to user and ask if they wish to
 use this model or recalibrate.
 If linear trend is not significant:
 Report result to user and ask them to recalibrate.

If linear polynomial is wanted:
 Fit recorded data with quadratic polynomial.
 If quadratic trend is significant:
 Plot data with quadratic polynomial values.
 If y-intercept value is significantly non-zero:
 Report result to user and ask if they wish to
 use this model or recalibrate.
 If y-intercept value is significantly zero:
 Report result to user and ask if they wish to
 use this model or recalibrate.
 If quadratic trend is not significant:
 Fit recorded data with a linear polynomial.
 If linear trend is significant:
 Plot data with linear polynomial values.
 If y-intercept value is significantly non-zero:
 Report result to user and ask if they wish to
 use this model or recalibrate.
 If y-intercept value is significantly zero:
 Report result to user and ask if they wish to
 use this model or recalibrate.
 If linear trend is not significant:
 Report result to user and ask them to recalibrate.

### sensor.py

This file includes code to obtain the voltage from the spectrophotometer using the Adafruit_MCP3008 package and converts the voltage to absorbance values.

### model.py

This file defines the calculations for fitting a polynomial model to the recorded data. It also includes code to make plots of the model’s values with the recorded data samples. The primary function is the train function that fits a model of the desired degree to data samples consisting of concentration and absorbance values.

Let $c_{i}$ and $a_{i}$ be the $i^{th}$ concentration and absorbance values, and $d$ be the degree of the desired polynomial. The model is defined to be

$$f\left( c_{n} \right)=w_{0}+w_{1} c_{n}+w_{2} c_{n}^{2}+\cdots+w_{d} c_{n}^{d},$$

where $w_{0},\ldots,w_{d}$ are the model’s coefficients and $d$ is 1, 2, or 3 for linear, quadratic, or cubic models. The coefficients are obtained by minimizing the mean-square error

$$\frac{1}{N}\sum_{n=1}^{N} \left( a_{n}-f\left( c_{n} \right) \right)^{2}.$$

The 95% confidence intervals for the coefficients $w_{0},\ldots,w_{d}$ are used to determine the significance of each term in the model. The user is notified of significant trends. For example, if the 95% confidence interval for $w_{0}$ does not include the value of 0, then the user is told that the model includes a non-zero y-intercept.

This file also defines a set of test data that can be used to test this application without being connected to the spectrophotometer.

### table.py

This code defines an interactive table with which data samples can be displayed and edited by the user.

### usb.py

This code is used to save data as a csv file to a drive connected to a USB port on the Raspberry PI.
